# PD-1 expression in transbronchial biopsies of lung transplant recipients is a possible early predictor of rejection

**DOI:** 10.3389/fimmu.2022.1024021

**Published:** 2023-01-10

**Authors:** Ilaria Righi, Valentina Vaira, Letizia Corinna Morlacchi, Giorgio Alberto Croci, Valeria Rossetti, Francesco Blasi, Stefano Ferrero, Mario Nosotti, Lorenzo Rosso, Mario Clerici

**Affiliations:** ^1^ Thoracic Surgery and Lung Transplantation Unit, Department of Cardio- Thoracic - Vascular Disease, Fondazione IRCCS Ca’ Granda Ospedale Maggiore Policlinico, Milan, Italy; ^2^ Division of Pathology, Fondazione IRCCS Ca’ Granda Ospedale Maggiore Policlinico, Milan, Italy; ^3^ Department of Pathophysiology and Transplantation, University of Milan, Milan, Italy; ^4^ Respiratory Unit and Adult Cystic Fibrosis Center, Internal Medicine Department, Fondazione IRCCS Ca’ Granda Ospedale Maggiore Policlinico, Milan, Italy; ^5^ Department of Biomedical, Surgical and Dental Sciences, University of Milan, Milan, Italy; ^6^ Don C. Gnocchi Foundation, IRCCS, Milan, Italy

**Keywords:** lung transplantation, chronic rejection, immunology, PD-1, immune checkpoint molecules

## Abstract

**Introduction:**

Chronic lung allograft dysfunction (CLAD) is the main cause of the reduced survival of lung transplanted (LTx) patients. The possible role of immune checkpoint molecules in establishing tolerance has been scarcely investigated in the setting of lung transplantation.

**Methods:**

We conducted a retrospective, observational pilot study on a consecutive series of transbronchial cryobiopsies (TCB) obtained from 24 patients during LTx follow-up focusing on PD-1, one of the most investigated immune checkpoint molecules.

**Results:**

Results showed that PD-1-expressing T lymphocytes were present in all TCB with a histological diagnosis of acute rejection (AR; 9/9), but not in most (11/15) of the TCB not resulting in a diagnosis of AR (p=0.0006). Notably, the presence of PD-1-expressing T lymphocytes in TCB resulted in a 10-times higher risk of developing chronic lung allograft dysfunction (CLAD), the main cause of the reduced survival of lung transplanted patients, thus being associated with a clearly worst clinical outcome.

**Discussion:**

Results of this pilot study indicate a central role of PD-1 in the development of AR and its evolution towards CLAD and suggest that the evaluation of PD-1-expressing lymphocytes in TCB could offer a prognostic advantage in monitoring the onset of AR in patients who underwent lung transplantation.

## Introduction

Despite the potency of currently available immunosuppressive drugs, the main factor limiting the success of transplantation is still immune-mediated rejection. Accurate human leukocyte antigen (HLA) matching reduces the occurrence of graft rejection, but in lung transplantation (LTx) the shortage of donors, time constraints, and lack of solid evidence make HLA typing not strictly required (1). HLA mismatch, together with primary graft dysfunction, the generation of donor-specific antibodies, postoperative infections, and suboptimal immunosuppression explain the high rate of LTx failure, as almost 50% of recipients reject their graft within 5 years of surgery (2).

Chronic lung allograft dysfunction (CLAD) is an irreversible decline of pulmonary function, and is the main cause of poor survival, low quality of life, and rising healthcare costs in LTx. CLAD can be the consequence of a single episode of acute rejection (AR) (3) and is characterized by two different phenotypes: bronchiolitis obliterans syndrome (BOS) and restrictive allograft syndrome (RAS) (4). BOS and RAS have different clinical features, radiological pattern, and outcomes, as RAS is associated with a significantly shorter survival and a worst response to therapy (5).

The gold standard diagnostic procedure for AR is transbronchial biopsy (TBB), performed either when rejection is suspected or for surveillance (6). The standardized morphologic evaluation of rejection, according to the International Society of Heart and Lung Transplantation (ISHLT) guidelines, defines histopathologic diagnostic criteria and grading, but it has a limited prognostic value towards clinical outcome and the likelihood to develop CLAD (7, 8). Furthermore, TBB evaluates immune cells present into lung tissues, but it cannot provide functional information on such cells (1). This is an important limit of TBB, as a more profound understanding of disease-associated immune alterations could lead to the development of new immune therapies that would result in clinical benefits (9). Moreover, tissue samples obtained with this technique could be inadequate to determine a pathological grade: in a large percentage of cases, diagnostic inadequacy is a consequence of crush artifacts, atelectasis and haemorrhage within alveoli (8, 9).

Transbronchial cryobiopsy (TCB), a fairly new technique largely used for the diagnosis of interstitial lung disease, allows to obtain larger samples with a higher number of alveoli and less artifacts compared to TBBs (10).

The modulation of antigen-specific immune responses and the creation of tolerance is the result of the interaction between several factors; immune checkpoint proteins have emerged as playing a fundamental role in these processes by inducing tolerance or apoptosis of antigens-specific lymphocytes. PD-1, in particular, is a pivotal player in down-regulating antigen-specific immune responses, the cornerstone of tolerance induction. The possible role of PD-1 in organ rejection has barely been analyzed (11, 12). In the setting of LTx we have recently shown that exhausted PD-1-expressing T lymphocytes and exhausted PD-1pos Treg T lymphocytes are significantly reduced in lungs that had been rejected within a RAS-type CLAD (13) suggesting that PD-1-expressing cells could be associated with a worst clinical outcome.

To shed further light on the possible role played by PD-1 in the establishment, or lack thereof, of lung graft tolerance, we analyzed the expression of this molecule in a consecutive series of transbronchial cryobiopsies (TCB) performed in patients who received bilateral lung transplantations.

## Patients and methods

### Study design

We performed a retrospective cohort study on patients who received LTx and underwent TCBs during follow-up. The pathologists (SF, GAC and VV) who analyzed TCBs were blinded to the clinical course of patients. Data collection was performed by three clinicians, two surgeons and one pneumologist, who reviewed all the cases. Patients’ overall survival from LTx and from TCB, responsiveness to high-dose steroids administration after diagnosis of AR, freedom from CLAD (months) as well as infections episodes were recorded for all patients. The median follow-up time was 39 months from LTx and 25 months from TCB (**Table 1**). The Hospital Institutional Review Board of Fondazione IRCCS Ca’ Granda Ospedale Maggiore Policlinico approved the study (ref. 1693/2018).

**Table 1:** Clinical characteristics of patients who underwent transbronchial cryobiopsy.

Patient	Sex	Age (years)	Disease	Time from LTx to TCB (months)	CSR	hAR	PD1	rHDS	AMR	CLAD	Alive (Yes/No)	FU from TCB (months)	FU from Tx
TCB #1	M	46	CF	18	Y	A2	Y	N	Y	Y	N	2	20
TCB #2	F	26	CF	14	Y	A1	Y	Y	Y	N	Y	27	42
TCB #3	M	60	CPFE	9	Y	A0	N	–	N	N	Y	27	36
TCB #4	M	29	CF	5	N	A0	N	–	N	N	Y	27	32
TCB #5	M	50	SS	14	Y	A1	N	Y	Y	N	Y	31	44
TCB #6	F	43	LAM	63	Y	A1	Y	Y	N	N	Y	29	80
TCB #7	M	64	LCH	18	N	A0	N	–	Y	N	Y	27	45
TCB #8	F	25	CF	23	Y	A1	N	N	N	Y	Y	26	49
TCB #9	F	44	CF	17	Y	A0	N	–	Y	N	Y	25	43
TCB #10	F	23	CF	6	N	A0	N	–	N	N	Y	25	30
TCB #11**	M	26	CF	11	Y	A1	Y	N	Y	Y	Y	6	17
TCB #12	M	30	CF	18	N	A0	N	–	N	N	Y	23	40
TCB #13	F	26	CF	17	N	A1	N	Y	N	N	Y	26	42
TCB #14	F	66	BR	15	N	A0	N	–	N	N	Y	24	39
TCB #15	M	35	CF	8	N	A0	N	–	N	N	Y	25	33
TCB #16	M	62	IPF	14	N	A0	N	–	N	N	Y	25	39
TCB #17	F	25	CF	9	N	A0	N	–	N	N	Y	25	34
TCB #18*	M	55	NSIP	12	Y	A0	N	–	N	N	N	10	22
TCB #19	F	27	CF	14	Y	A2	Y	N	N	Y	Y	12	26
TCB #20	M	26	CF	25	Y	A3	Y	N	Y	Y	N	8	33
TCB #21	F	32	CPFE	2	Y	A1	Y	N	N	Y	Y	41	43
TCB #22	F	41	CF	73	Y	A2	Y	N	N	Y	Y	31	104
TCB #23*	M	50	HP	6	Y	A2	N	N	Y	N	N	4	10
TCB #24	M	35	CF	17	Y	A1	Y	Y	N	N	Y	29	46

AMR, Antibodies Mediated Rejection; BR, Bronchiectasis; CF, Cystic Fibrosis; CPFE, Combined Pulmonary Fibrosis and Emphysema; CSR, Clinical Suspicion of Rejection; FU, Follow-up; HP, Hypersensistivity Pneumonitis; hAR, histologic Acute Rejection; IPF, Idiopathic Pulmonary Fibrosis; LAM, Lymphangioleiomyomatosis; LCH, Pulmonary Langerhans Cell Granulomatosis - Histiocytosis X; NSIP, Non-Specific Interstitial Pneumonia; rHDR, responder to High Dose Steroids; SS, Systemic Sclerosis.

* these patients died from a cause not related to CLAD.

** this patient underwent re-transplant for RAS at seven months after first LTx.

### Patients

A consecutive series of 24 LTx patients who underwent TCB between 2018 and 2019 was analyzed. Patients’ epidemiological characteristics are reported in **Table 1**. Thirteen patients (58%) were male; median age was 35 years (95% C.I. 26.7-47). TCBs were performed in fifteen cases (62%) for clinical suspicion of rejection whereas nine patients underwent cryobiopsies for surveillance purpose. The median time interval between transplantation and TCB was 14 months (95% C.I. 9 - 17.2 months). Thirteen patients (54%) had evidence of histological grade A acute rejection (hAR) on TCB, while eight (33%) had a diagnosis of antibody mediated rejection (AMR) (7, 14). Median follow-up after TCB was 12 months (95% C.I. 12.0-13.3 months); seven patients (29%) developed CLAD and six of them (25%) died, one of them from intractable acute antibody mediate rejection (patients #23). All patients received the standard-of-care immunosuppressive therapy (i.e. corticosteroids, tacrolimus and azathioprine). The thirteen patients with a diagnosis of hAR also received pulsed corticosteroids (10 mg/kg), which restored optimal pulmonary function in five cases (38%). The TCB technique is described elsewhere (10, 15).

### Immunohistochemical analysis

Representative 4-μm-thick sections were cut from each block and stained with a PD1/CD279 specific antibody (clone NAT105; Ventana Medical Systems, part of Roche Diagnostics, Monza, Italy), as previously described (13). Positive and negative controls were included in each experiment. All slides were counterstained with hematoxylin and digitalized using Aperio scanner at 40x magnification (Leica Microsystems). Presence of PD1-positive lymphocytes was performed manually and a slide was judged negative (no staining in lymphocytes), with individual positive cells scattered in the lung parenchyma (1-5% of the lymphocytic infiltrate), or positive if positive lymphocytes were >5%. This cut-off was determined using a series of 8 CLAD lungs and represent the best score able to sort RAS lungs from BOS lungs (**Supplementary Figures 1A, B**). For statistical purposes, negative TCBs and samples with ≤5% of PD1-positive lymphocytes were grouped together.

### Broncho-alveolar lavage cytology

Giemsa-stained slides obtained from cell-blocks were available for 19 patients. Two cases were excluded due to the poor cellularity. A cell count was performed by sorting out the histiocytes and the epithelial cells. Percentage of lymphocytes, neutrophils, eosinophils and basophils was then assessed as the average value of two counts (100 cells each) using a cell counter.

### Statistical analyses

Collected clinical data were summarized with absolute and percentage frequencies or median and range or 95% confidence interval, as appropriate. IHC and cytological data were presented as percentages of positive cells and summarized using individual value plots with median and interquartile range (IQR), unless otherwise specified. Samples were compared using the non-parametric Mann Whitney U test. For categorical analyses, the number of patients in each category is shown, and data were analyzed using Chi-square or Fisher exact test as appropriate. Finally, a binary diagnostic test related to the composite endpoint consisting of BOS and RAS was performed to compute sensitivity, specificity and the risk ratio values.

After dichotomizing PD-1 using the threshold of 5%, we evaluated the characteristics of PD-1 as binary diagnostic test. Analyses were performed using the MedCalc (MedCalc Software Ltd, Ostend, Belgium) or R studio (version 3.2.2) and charts were generated with GraphPad Prism software (San Diego, CA, USA).

## Results

### PD-1 expression in transbronchial cryobiopsies

PD-1 expression was analyzed in TCBs obtained in a consecutive series of 24 LTx patients; this parameter was not scored as a continuous variable because of the limited tissue available in biopsies. As a cut-off value we used 5% of positive lymphocytes, which is the value that best discriminates RAS from BOS CLAD (**Supplementary Figures 1A, B**).

**Figure 1:**
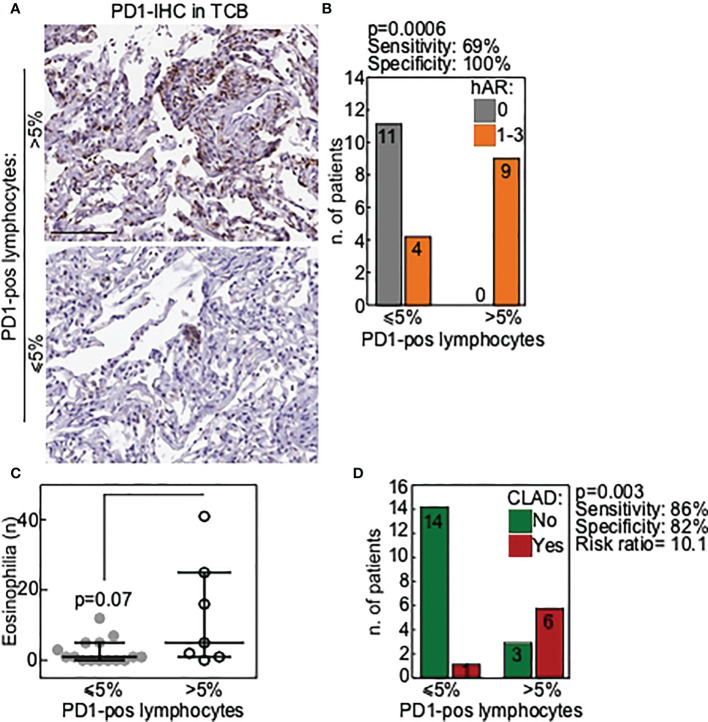
PD1 is a marker of poor allograft outcome in transbronchial cryobiopsies of LTx patients. **(A)** Representative images of TCB with a lymphocytic infiltrate positive for PD1 (>5% of positive lymphocytes; upper panel) or negative (≤5% of positive lymphocytes; lower panel). Scale bars, 100 μm. **(B)** Correlation of PD1-positive TCB with histological diagnosis of acute rejection (hAR grade A). *P* value is from Fisher exact test. **(C)** Presence of eosinophilia in BAL was correlated with PD1-positive TCB. Each dot is a case and lines indicate median with IQR. *P* value is from Mann-Whitney U test. **(D)** Correlation of PD1-positive TCB with occurrence of chronic lung allograft dysfunction (CLAD) during follow-up. *P* value is from Fisher exact test.

Results showed that PD-1-expressing lymphocytes (**Figure 1A**) were present in 9/24 (38%) TCBs. No difference in PD-1 expression could be detected according to patients’ diagnosis (CF *vs* others). Notably, hAR grade A1-A3 was diagnosed in all the nine (100%) TCBs where PD-1-expressing immune cells were detected (**Figure 1B**). PD-1-expressing lymphocytes were absent in the remaining 15/24 (62%) TCBs, and hAR was diagnosed in only 4/15 (27%) of these cases. A histological diagnosis of acute rejection was not formulated in any of the 11/15 remaining PD-1-negative TCBs (p=0.0006; **Figure 1B**). Statistical analyses indicated that the presence of PD-1-expressing cells in TCB has a sensitivity of 0.69 and a specificity of 1 toward the presence of hAR (**Figure 1B**).

In the attempt to correlate PD-1 expression with other biomarkers suggested to have a predictive value toward lung rejection, BAL from all the individuals enrolled in the study was analyzed next. Results showed that eosinophils were increased in the BAL of those patients in whom PD-1-expressing-lymphocytes were present (**Figure 1C**). This result approached but did not reach statistical significance (p=0.07), likely because of the limited number of the available samples.

We finally verified whether the presence of PD-1-expressing cells in TCBs was associated with a higher likelihood of developing CLAD. This was indeed the case, as CLAD developed in 7/24 (33%) patients and PD-1-expressing cells were detected in the TCBs of 6/7 (86%) of these patients (p=0.003; **Figure 1D**).

Results of statistical analyses showed that PD-1-expressing cells in TCB has a sensitivity of 0.86 and a specificity of 0.82 toward the likelihood of developing CLAD, with a positive predictive value of 0.67, and a negative predictive value of 0.93. Overall, the presence of PD-1-expressing cells in the TCB results in a 10 times higher risk of developing CLAD (**Figure 1D**).

### Clinical follow-up

**Figure 2:**
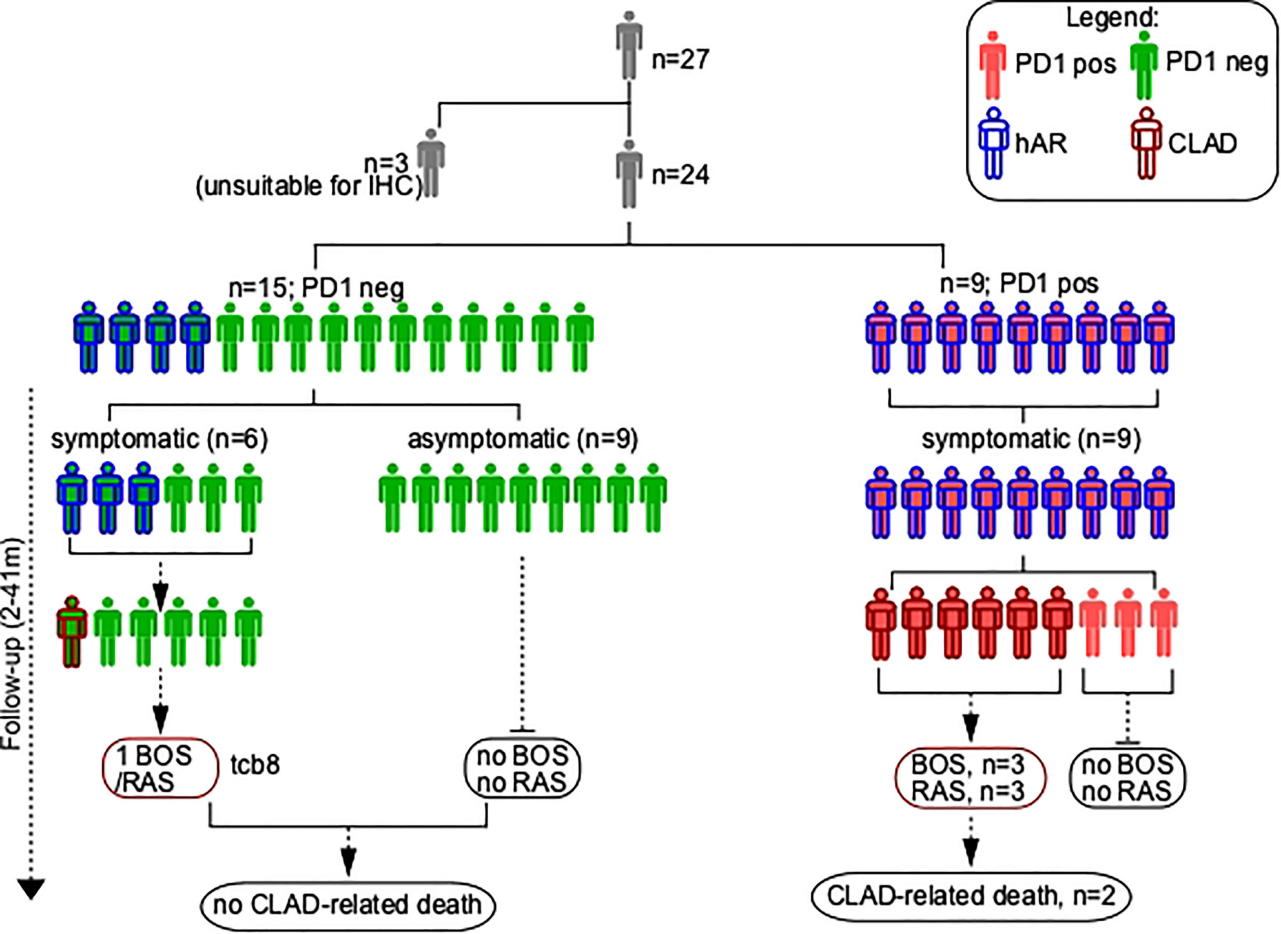
Schematic of TCB patients’ series. hAR, Histological diagnosis of acute rejection (grade A); CLAD, chronic lung allograft dysfunction; PD1, Programmed cell death protein 1; BOS, bronchiolitis obliterans syndrome; RAS, restrictive allograft syndrome.

Median clinical follow-up from TCB was 25 months (2-41 months). At the one-year follow-up time-point, an optimal graft function characterized 12 of the 15 patients (80%) in whom PD-1-expressing T lymphocytes were absent in the initial TCB (**Figure 2**). The three exceptions being as follows: 1) EBV (Epstein-Barr Virus) infection and BOS with a favorable clinical presentation (good response to high dose steroids, no need for oxygen therapy; patient #8); 2) death for EBV- related cerebral lymphoma (patient #18); and 3) death for acute humoral rejection (patient #23). Notably, this indicates that in none of these three patients a cell-mediated graft rejection was observed.

Histological AR was diagnosed at the follow-up time point in 4 of the 15 patients (26%) in whom PD-1-expressing T lymphocytes were absent in the initial TCB. hAR was clinically mild in patients #5, #8, and #13 (optimal FEV1 response to pulsed steroids (10mg/Kg); no need for oxygen therapy); the fourth case was patient TCB#23 (see above). Clinically relevant graft infections were not detected in any of these four individuals.

The follow-up of the 9 patients whose TCB stained positive for PD-1 was drastically different, as all these patients had an unfavorable clinical outcome. Thus, 1) patients #1, #19, #22 (hAR grade 2) and #20 (hAR grade 3) developed a graft failure that evolved in CLAD and led to the death of patients #1 and #20; 2) patient #11 (hAR grade 1) had a critical loss of graft function that led to retransplantation; and 3) patient #21 (hAR grade 1) developed BOS grade 4. The remaining three patients (#2, #6, #24) in whom PD-1-expressing T lymphocytes were present in the baseline TCBs developed a grade 1 hAR that did respond to high dose steroids and did not evolve to CLAD.

Two different TCBs, performed at 8-months interval, were available for patient #24. In the initial sample neither PD-1-expressing T lymphocytes nor hAR were present. The second TCB was performed because a graft infection occurred; notably, in this case PD-1-expressing T lymphocytes were present and a hAR grade 1 was diagnosed.

## Discussion

Immune tolerance is mediated by a number of complex immunological mechanisms in which the family of proteins collectively known as checkpoint molecules plays a pivotal role (16). In this retrospective study we analyzed the expression of one of these molecules, PD1, in the setting of LTx. PD-1 is an inhibitory receptor that is expressed by activated T cells and regulates T cell effector functions in infection, cancer and autoimmunity, playing a fundamental role in immune tolerance (16–20). Results herein show that the presence of PD-1-expressing lymphocytes identified the vast majority of transbronchial biopsy (TCBs) where a histological diagnosis of acute rejection was formulated. Notably, the presence of PD-1-expressing lymphocytes in TCBs was also predictive of a significantly higher likelihood of developing CLAD, and of a significantly worst clinical scenario at the one-year follow up.

The initial question we addressed was whether the detection of PD-1- expressing lymphocytes could have a prognostic value in TCBs performed during routine follow-up in LTx patients, hence addressing the question of whether the expression of immune checkpoints could predict allograft rejection. Results of this pilot study indeed confirmed our hypothesis. Thus, a histological AR diagnosis was present in all the cases in which PD-1-expressing lymphocytes were detected, and, even more strikingly, PD-1-expressing lymphocytes were absent in all the patients without a diagnosis of acute rejection. Notably, the presence of PD-1-expressing cells in TCB was also associated with a 10-times higher risk of developing CLAD. This was confirmed by the longitudinal follow-up of the patients, which showed that the presence of PD-1-expressing cells in TCB correlated with a clearly worst clinical LTx outcome.

Possible correlations with other biomarkers (21–27) suggested to be associated with graft rejection were sought; results showed that eosinophils were increased in BAL from PD-1-positive TCB. Eosinophils are known to damage the lung by degrading connective tissue and injuring epithelial and microvascular structure. Several clinical studies indicate that increased eosinophils in BAL associates with AR and a worse outcome in LTx recipients and is a risk factor for CLAD development (28). This result offers further support to the hypothesis that PD-1 expression could be a negative prognostic marker in LTx.

PD-1 is a membrane protein that controls the magnitude of T-cell responses. Through the ligation of PD-L1, its main receptors, PD-1 modulates tolerance and inhibits T-cell mediated immunity, thus playing a fundamental role in immune responses (16–20, 29–33). PD-1 can be expressed by exhausted T cells in situations of chronic antigenic stimulation, and PD-1-expressing exhausted cells are known to undergo apoptotic cell death (24). This is a negative prognostic index in chronic infections and cancer, where disease progression is associated with the waning of immune responses. Loss of antigen-specific immune responses should nevertheless be a positive factor in transplant immunology, as it favors the generation of self-tolerance. It is thus apparently counterintuitive that the detection of these cells results in a worst clinical outcome in LTx. Further, whether PD1 expression is a consequence, or a cause of allograft rejection is still unknown and functional studies to understand the mechanisms of immune checkpoints fine-tuning in transplanted organs are needed.

Our results, nevertheless, are reinforced by recent data showing that the presence of PD-L1-expressing cells is predictive of organ rejection in patients who received heart transplantation (34, 35). Even more recently, exhausted PD-1-expressing T lymphocytes and exhausted PD-1 positive Treg T lymphocytes were shown to be significantly reduced in lungs that had been rejected within a RAS-type CLAD, suggesting that PD-1-expressing cells results in a worst clinical outcome in LTx (13). Altogether these data may provide insights into molecular mechanisms adopted by the immune to fine-tune between allograft tolerance and rejection. Consecutive samples from the same patients during post-LTx surveillance may shed light on the timing of immune checkpoint molecules activation and repression.

The limited sample size and the type of transbronchial biopsy required to obtain sufficient material for analysis are clear limitations of this study: cryobiopsy is not the most common method to monitor LTx patients, even if it is the one that provide ampler tissue samples. Lastly, immunophenotyping of PD1 expressing lymphocytes in transbronchial biopsies should be performed in future studies to obtain functional and biological clues. The need for further analyses notwithstanding, results herein suggest that PD-1 expression is an early, specific, and sensitive biomarker of rejection in surveillance TCBs and indicate that the use of PD-1 immunohistochemistry staining could improve the diagnosis and grading of rejection. These results could also be useful in the design of novel immune-mediated therapies to possibly prevent or modulate graft rejection in LTx.

## Data availability statement

The raw data supporting the conclusions of this article will be made available by the authors, without undue reservation.

## Ethics statement

The studies involving human participants were reviewed and approved by The Hospital Institutional Review Board of Fondazione IRCCS Ca’ Granda Ospedale Maggiore Policlinico approved the study (ref. 1693/2018). The patients/participants provided their written informed consent to participate in this study.

## Author contributions

IR and MC conceived the study and co-wrote the manuscript; VV designed the experiments, performed the immunohistochemical analyses, and co-wrote the manuscript; LM and VR were responsible for the clinical follow up of patients; GC performed immunohistochemical analyses; FB and SF designed the study and co-wrote the paper; IR, MN and LR performed surgical procedures and co-wrote the manuscript. All authors contributed to the article and approved the submitted version.
