# Supplementary material for: PD-1 expression in transbronchial biopsies of lung transplant recipients is a possible early predictor of rejection
Source: Front Immunol. 2023 Jan 10;13:1024021. doi: 10.3389/fimmu.2022.1024021 (PMC9871480; doi:10.3389/fimmu.2022.1024021)
Supplement: Supplementary file 1 [file DataSheet_1.docx]

**PD-1 Expression in Transbronchial Biopsies of Lung Transplant Recipients**

**is a Possible Early Predictor of Rejection**

Ilaria Righi, Valentina Vaira, Letizia Corinna Morlacchi, Giorgio Alberto Croci, Valeria Rossetti^3^, Francesco Blasi, Stefano Ferrero, Mario Nosotti,

Lorenzo Rosso, Mario Clerici.

**Supplementary Figure**

**
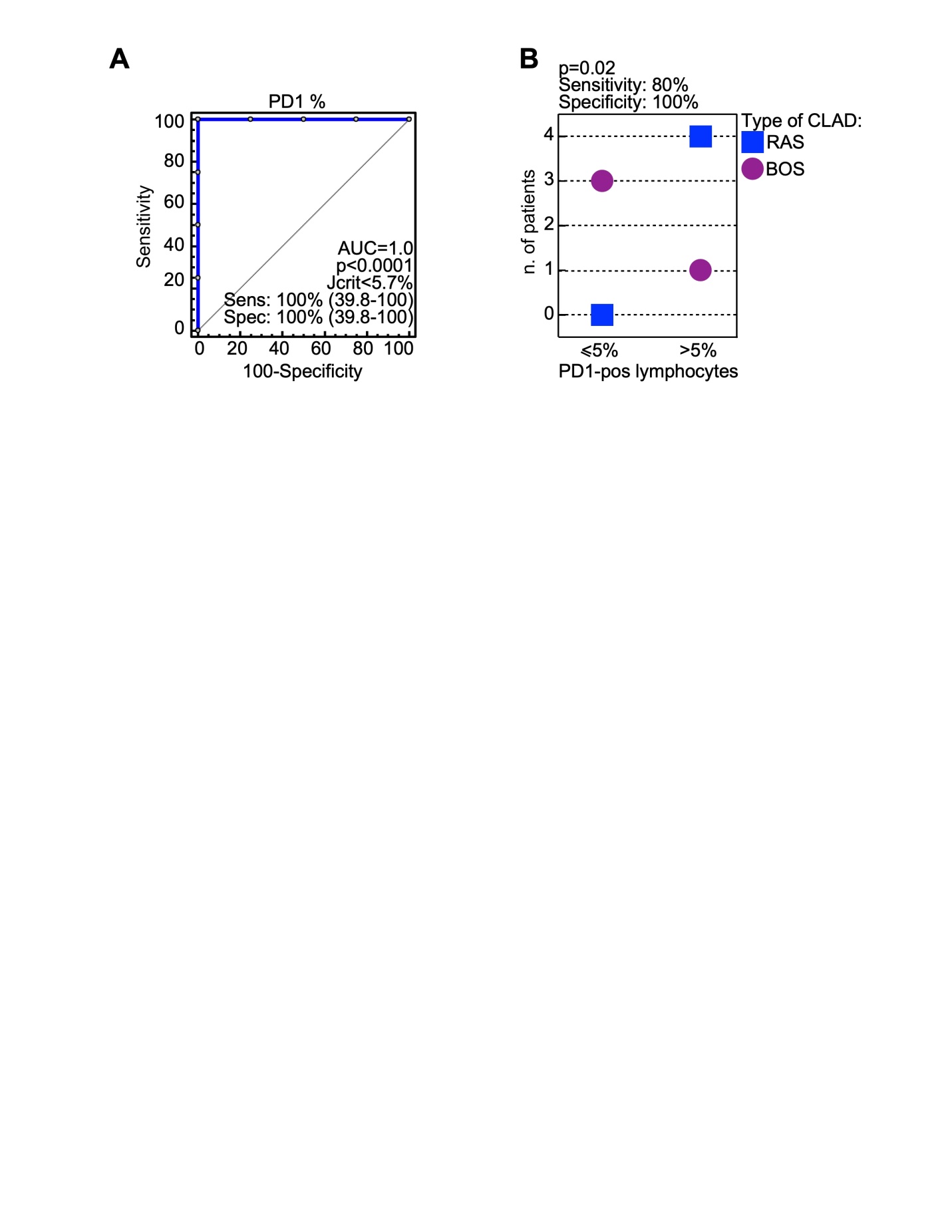
**

**Suppl. Figure 1. Identification of the optimal cut-off for PD1 expression.** The ROC analysis with the Youden criterion (A) was used to identify the optimal cut-off to differentiate RAS from BOS CLAD. Performance of the classification is shown in panel B.
